# Batroxobin accelerated tissue repair via neutrophil extracellular trap regulation and defibrinogenation in a murine ischemic hindlimb model

**DOI:** 10.1371/journal.pone.0220898

**Published:** 2019-08-16

**Authors:** Haruchika Masuda, Atsuko Sato, Tomoko Shizuno, Keiko Yokoyama, Yusuke Suzuki, Masayoshi Tokunaga, Takayuki Asahara

**Affiliations:** 1 Department of Physiology, Tokai University School of Medicine, Isehara, Kanagawa, Japan; 2 Department of Regenerative Medicine Science, Tokai University School of Medicine, Isehara, Kanagawa, Japan; 3 Department of Research and Education Support Center, Tokai University School of Medicine, Isehara, Kanagawa, Japan; Medical University Innsbruck, AUSTRIA

## Abstract

Batroxobin, isolated from *Bothrops moojeni*, is a defibrinogenating agent used as a thrombin-like serine protease against fibrinogen for improving microcirculation. Here, we investigated whether, and if so, how batroxobin restores ischemic tissue injury in terms of anti-inflammatory effects. In an *in vitro* flow cytometry assay for human neutrophil extracellular traps (NETs), batroxobin (DF-521; Defibrase) inhibited human NETs induced by tumor necrosis factor-α (TNF-α) in the presence of human fibrinogen. Next, the effect of batroxobin was investigated by immunohistochemistry of the anterior tibial muscle (ATM) in an ischemic hindlimb model using C57BL/6J mice intraperitoneally injected with DF-521 versus the saline control. NETs and fibrinogen deposition in the ischemic ATM decreased in DF-521-treated mice on day 2 after ischemia. Meanwhile, reverse transcription-quantitative PCR assay of the ischemic ATM unveiled continuous downregulation in the expression of the genes; *Tnf-α* and nitric oxide synthase2 (*Nos2*) with hypoxia-inducible factor-1α (*Hif-1α*) and vascular endothelial growth factor-a (*Vegf-a*) from day 3 to day 7, but the upregulation of arginase-1 (*Arg-1*) and placental growth factor (*Plgf*) with myogenin *(Myog)* on day 7. Daily intraperitoneal DF-521 injection for the initial 7 days into mice with ischemic hindlimbs promoted angiogenesis and arteriogenesis on day 14. Moreover, DF-521 injection accelerated myofiber maturation after day 14. Laser doppler imaging analysis revealed that blood perfusion in DF-521-injected mice significantly improved on day 14 versus the saline control. Thus, DF-521 improves microcirculation by protecting NETs with tissue defibrinogenation, thereby protecting against severe ischemic tissue injury and accelerating vascular and skeletal muscular regeneration. To our knowledge, batroxobin might be the first clinically applicable NET inhibitor against ischemic diseases.

## Introduction

Batroxobin (Defibrase) is a thrombin-like serine protease extracted from the venom of the snake *Bothrops moojeni*, known as the Brazilian lancehead, and it exerts defibrinogenating and anti-inflammatory effects [1–4]. The defibrinogenating effect of batroxobin improves microcirculation by reducing blood viscosity, as reported in experimental studies on cochlear blood flow in guinea pigs [5] and middle cerebral artery blood flow in humans [6]. Considering the effect of defibrinogenation in improving peripheral circulation [7,8], batroxobin is clinically applied to manage conditions like vibration disease [9], sudden deafness (in Japan) [10,11], stroke, and transient ischemic attack (in China) [12,13].

Regarding batroxobin-inducing defibrinogenation, Wang et al. reported that thrombin first releases fibrinopeptide A and then fibrinopeptide B from fibrinogen (Fgn). The release of both the peptides triggers a polymerizing reaction for fibrin monomers to form a polymer with a tight linking ability.

Meanwhile, batroxobin cleaves fibrinopeptide A, but not ibrinopeptide B, from Fgn, which triggers the des-A fibrin monomer to form a polymer with a looser linking ability than that of the fibrin polymer. The des-A fibrin polymer evokes easy degradation by plasmin in the fibrinolytic system, and the degraded product is finally excluded by the reticuloendothelial system [14], resulting in the actualized defibrinogenation.

Although the anti-inflammatory effect of batroxobin was reported almost three decades ago [3,4], the underlying molecular or cellular mechanism has yet to be detailed. Batroxobin injection has been reported to result in a dose-dependent decrease in the Fgn concentration in rat plasma. It has also been reported to improve the pathogenic inflammation status [3] in animal models, brain edema [15], immune complex-mediated glomerulonephritis [16], autoimmune encephalomyelitis [17], and spinal cord injury [18]. As Fgn is well-known as a critical agent inciting inflammation [19–21], its anti-inflammatory effects are currently attributed to the rapid compensation of plasma Fgn by des-A fibrin polymer production under the inflammatory milieu.

Owing to the anti-inflammatory effect of defibrinogenating batroxobin, we must also consider the effect of batroxobin on the behavior of inflammatory blood cells, especially neutrophil extracellular traps (NETs) [22], as a scaffold of microthrombosis to enhance inflammation, i.e., “microthrombotic inflammation” [23]. Under inflammation, blood flow impeded by fibrinogenesis leads to blood hyperviscosity, thereby stimulating thrombogenicity. NETs not only cause inflammatory tissue injury via extracellular release of the DNA-histone complex with cytoplasmic proteinases (myeloperoxidase [MPO], elastase, etc.) but also provide a platform for thrombogenesis [24]. For instance, Fgn colocalizes with coagulation factors in NETs [25], which accelerate clot formation and delay its lysis [26,27].

Considering these accounts, the anti-inflammatory effect of batroxobin in terms of the impact on NET formation involved in defibrinogenation remains to be elucidated. Herein, we hypothesized that batroxobin might regulate NET formation and defibrinogenation, perhaps leading to a reduction in inflammatory tissue damage. The present study aimed to investigate the effect of batroxobin on NETs and the successive inflammation using a mouse ischemic hindlimb model.

## Materials and methods

### Abbreviations

The abbreviations with the corresponding complete names are listed in **S1 Table**.

### Reagents and items

The reagents and items used in the experiments are listed in **S2 Table**.

### Isolation of human neutrophils

Experiments using human samples were performed with institutional approval and according to the guidelines of the Clinical Investigation Committee at Tokai University School of Medicine (Institutional Review Board [IRB] No. 13R228). Ten to fifteen milliliters of human peripheral blood from healthy unrelated volunteers 20–50 years of age was aspirated by a 20 mL heparinized syringe, with their written consent. Human neutrophils were isolated using PolymorphoPrep density gradient media (#1114683; PROGEN Biotechnik GmbH, Heidelberg, Germany) according to the manufacturer’s protocol. In brief, 15 mL aspirated blood was laid over an equivalent volume of density gradient medium in a 50 mL conical tube and centrifuged at 480 *×g* and 20°C for 30 min. After the upper layer of mononuclear cells was aspirated, neutrophils in the lower layer were transferred to a 15 mL conical tube with 10 mL Ca^2+^, Mg^2+^ free Hank’s balanced salt solution (#085–09355; FUJIFIM Wako Pure Chemical, Osaka, Japan). After the cell pellet was harvested by centrifugation at 400 *×g* and 20°C for 20 min, it was hemolyzed with 2 mL diluted BD Pharm Lyse lysing buffer (10 x) (#55589; BD Biosciences, San Jose, CA, USA) with sterilized distilled water at room temperature (RT) for 5 min. Neutrophils were harvested by centrifugation at 300 *×g* and 4°C for 10 min, after making up the volume to 15 mL with phosphate buffer saline (PBS)- 2 mM ethylenediaminetetraacetic acid (EDTA) in the tube. The isolated neutrophil pellet was suspended with 2 mL of 1% (v/v) fetal bovine serum (FBS)/RPMI-1640 (#187–02021; FUJIFIM Wako Pure Chemical) including penicillin–streptomycin antibiotics (#P4333; Sigma-Aldrich, St. Louis, MO, USA) in a 1:100 ratio. The suspended neutrophils were filtered through the 35 μm nylon mesh of a cell-strainer cap filter attached to a 5 mL polystyrene round bottom tube (# 352235; Corning, Oneonta, NY, USA).

Finally, the suspended cells from the cellular fraction in the lower layer was identified to comprise neutrophils on the basis of high positivity rates for neutrophil surface markers-99.8% for CD16, 99.5% for CD11b, and 62.3% for CD66b- using flow cytometry (**S2 Table, S1 Fig**). The cells in the lower layer as neutrophils were applied for each *in vitro* assay.

### Flow cytometry analysis of human NETs *in vitro*

The assay was performed by using flow cytometry, as recently reported [28]. At first, 500 μL of each conditioned medium of 1% (v/v) FBS/RPMI-1640 with human Fgn (hFgn) (2 mg/mL) (#F4883; Sigma-Aldrich) and recombinant human tumor necrosis factor-α (rhTNF-α) (100 ng/mL) (#AF-300-01A, PeproTech, Rocky Hill, NJ) in the presence or absence of DF-521 (0.4 BU/mL) (Tobishi Pharmaceutical Co., Ltd., Tokyo, Japan) was applied per well of a 24-well suspension plates (#662102; Greiner, Wagenseilgasse, Wien, Austria). The isolated neutrophils were suspended in 1% (v/v) FBS/RPMI-1640 medium and seeded at 1 × 10^6^ cells/500 μL per well with 500 μL of each conditioned medium. The neutrophils in each conditioned medium were incubated in a 5% CO_2_ incubator at 37°C for 4 h. Each of the conditioned media containing non-adherent neutrophils were first harvested into 15 mL conical tubes. The adherent neutrophils were also harvested by pipetting with 800 μL PBS-2 mM EDTA into the corresponding tubes and were completely assembled by repeating the procedure twice. The harvested neutrophils were centrifuged at 220 *×g* and 4°C for 15 min. The pellets were suspended in 200 μL flow cytometry buffer (PBS-2 mM EDTA containing 0.1% [v/v] FBS). The neutrophils suspended in each condition were again filtered through a new similar filter put on a 1.5 mL capless graduated microcentrifuge tube (#NC-509-GRD-Q; Quality Scientific Plastics, San Diego, CA, USA). After the addition of 2 μL True Stain Monocyte Blocker (#426101; BioLegend, San Diego, CA, USA), the cell suspension was divided into two 100 μL aliquots. The conditioned neutrophils in each 100 μL aliquot of flow cytometry buffer were stained with 2 μL of mouse anti-human MPO antibody–FITC conjugate (#ab11729; clone 2C7; Abcam, Cambridge, UK) or an isotype control of BD Pharmingen Mouse IgG1κ–FITC conjugate (#555909; clone MOPC-21; BD Biosciences) at 4°C for 30 min. After neutrophils were washed in each aliquot twice with 800 μL flow cytometry buffer at 800 *×g* and 4°C for 2 min, the stained neutrophils were resuspended in 200 μL flow cytometry buffer.

To stain the extracellular DNA of NETs, Sytox Orange (SO) (#S-11368; Molecular Probes, Eugene, OR, USA) was added at the final concentration of 5 μM after diluting with flow cytometry buffer, and incubated at RT for 5 min. The stained neutrophils were subjected to flow cytometry in a BD FACSVerse flow cytometer (Becton Dickinson, Franklin Lakes, NJ, USA). The data were analyzed by FlowJo software (Ver10.4.1; Tomy Digital Biology CO., Ltd., Tokyo, Japan). The assays were repeated with three different volunteers (one volunteer per day) to obtain experimental replicates.

The protocol is indicated in the following online site; http://dx.doi.org/10.17504/protocols.io. [dx.doi.org/10.17504/protocols.io.3zzgp76].

### Scanning electron microscopy

Each sterilized 13 mm diameter coverglass (#C1100; Matsunami Glass Ind., Ltd., Osaka, Japan) was plated in single wells in a 24-well suspension plate. Each coverglass per well was evenly coated with 250 μL of 0.01% (w/v) poly-l-lysine solution (#P4707-50ML; Sigma-Aldrich) and incubated at RT for 5 min. After the solution was removed, the coverglass was rinsed with 1 mL sterile distilled water three times and dried for 3 h. Neutrophils in each of the conditioned media were adjusted in wells with poly-l-lysine-coated coverglasses according to the same protocol as the former assay and incubated for 90 min in a 5% CO_2_ incubator. The incubated neutrophils were processed for observation by scanning electron microscopy (SEM). The samples on coverglasses in 24-well plates were fixed with 2.5% (v/v) glutaraldehyde for 2 h and then with 1% (w/v) osmic acid for 30 min. Next, dehydration was performed using serially increasing concentrations of ethanol for 10 min at each concentration, including a final hydration with dehydrated ethanol three times. Then, the samples were transferred into *t*-butyl alcohol and frozen in a freezer. The frozen specimens were freeze-dried in a JFD-310 freeze-drying device (JEOL Ltd., Tokyo, Japan). The samples were coated with osmium by using an osmium coater (Neoc-Pro; Meiwafosis Co., Ltd., Tokyo, Japan) and observed at 15 kV using SEM (JSM-6510LV; JEOL).

### Animals

The protocols of the animal experiments were approved by the Institutional Animal Care and Use Committee of the Isehara Campus, Tokai University School of Medicine (Institutional Reference Nos. 171091 and 182051).

Eight-week-old male C57BL/6J mice were purchased from Charles River Laboratories Inc. (Yokohama, Japan) and acclimatized for 2 weeks at a 12 h light-dark cycle. The mice were fed with standard laboratory chow (CLEA Rodent Diet CE-2; CLEA Japan, Inc., Tokyo, Japan) and water *ad libitum*; wood enrichment was also applied. The animal experiments were performed in line with the regulations for the improvement of animal welfare, as well as “replacement, reduction, and refinement” (3Rs).

### Mouse hindlimb ischemia model

To induce hindlimb ischemia and measure hindlimb blood perfusion, mice were subjected to inhalation anesthetization using 1.5~2.0% (v/v) isoflurane (isoflurane inhalation solution, 1 mL/mL, Pfizer Inc., New York, NY, USA). Mice at the age of 10 weeks underwent surgery to develop the hindlimb ischemia model as previously described [29].

In brief, under anesthetization (which was checked by the lack of mouse response to pinching of the skin with forceps), an incision in the skin of the left hindlimb at the distal portion from the inguinal ligament was made.

After the removal of subcutaneous fat tissue, the femoral artery was first dissected from the femoral vein with a cotton swab. By using 6–0 surgical sutures, the dissected artery was ligated at the distal portion from the branch of the lateral circumflex femoral artery and at the proximal portion from the bifurcation of the popliteal and saphenous arteries (**S2 Fig**). All the arterial branches between the two ligated portions were also ligated with their veins to run in parallel. The ligated blood vessels were excised. The surgical window of the skin was closed with a surgical stapler. Subsequently, they were subcutaneously injected 0.1 mg/kg of Buprenorphine Hydrochloride (Repetan; Otsuka Pharmaceutical Co., Ltd., Tokyo, Japan) for analgesia, put back in their cages, and warmed up under a infrared lamp to aid faster recovery. The intraperitoneal administration of batroxobin (DF-521) adjusted with saline was started at a dose of 30 BU/kg per head daily from 2 h or 24 h after the surgery. The mice were monitored for fluffing or hunched posture every day for the next three days after surgery and thereafter once every three days to assess the effect on health and well-being. In fact, after surgery, no aggravated health issue was found in the mice in any group.

### Blood perfusion analysis

Blood perfusion analysis was performed as described elsewhere [29]. Under the similar inhalation anesthetization at making the hindlimb ischemia model, the mice were put on an infrared warming pad for the blood perfusion analysis.

Then, laser Doppler perfusion imaging (LDI; Moor Instruments, Ltd., Devon, UK) was used to record serial blood flow measurements before ischemia was induced, and on day 0, day 4, day 7, and day 14 after ischemia; these data were analyzed using Moor LDI Main software (Laser Doppler Imager ver 5.3; Moor Instruments, Ltd.).

The blood flow in the region of interest of ischemic and contralateral limbs in each mouse on an infrared warming pad was measured by laser Doppler perfusion imaging, and the blood flow ratio of ischemic versus contralateral hindlimbs was calculated.

### Pretreatment at the and sampling after inducing ischemia

In the pretreatment procedure before sacrifice, when sampling the anterior tibial muscle (ATM) in each assay, high-dose pentobarbital sodium (200 mg/kg body weight Somnopentyl; Kyoritu Seiyaku Co., Ltd., Tokyo, Japan) was intraperitoneally injected into the hindlimb ischemia model mice. When mice showed no response to pinching the skin with forceps, whole blood was aspirated by heart puncture with a heparinized 22G butterfly needle (Terumo Co., Tokyo, Japan), followed by perfusion with 25 mL EDTA-free PBS.

### Reverse transcription-quantitative PCR of murine ATM

Immediately after the pretreatment, the ATM was cut out, immersed in 700 μL RNAlater Stabilization Solution (#AM7020; Invitrogen, Carlsbad, CA, USA) in a 1.5 mL tube, and incubated at 4°C overnight. The next day, the ATM in RNAlater was stored at -80°C until total RNA isolation. Total RNA was isolated by TRIzol (#15596–018; Invitrogen), and genomic DNA was digested by DNase I treatment (#18068; Invitrogen) at 37°C for 15 min. DNase I-treated total RNA was purified by phenol extraction and ethanol precipitation. One hundred nanograms of purified total RNA was used for cDNA synthesis with the SuperScript VILO cDNA synthesis kit (#11754250; Invitrogen). The cDNA mixture was diluted 10-fold after first-strand cDNA synthesis. Using ABI Prism 7700 (Applied Biosystems, Waltham, MA, USA), reverse transcription-quantitative PCR for diluted cDNA was performed using EagleTaq Master Mix (#5529085190; Roche, Mannheim, Germany), 0.3 mM of forward and reverse primers used for cDNA amplification, and 0.25 mM of TaqMan probe (Applied Biosystems) according to the manufacturer’s protocol. The relative mRNA expression was calculated for each gene by the 2^-ΔΔCT^ method with normalization against mouse 18S ribosomal RNA (18S rRNA). All primers and TaqMan probes used are listed in **S2 Table**.

### Histological assessment

After the pretreatment, the ischemic hindlimbs of the mice were excised out and fixed with 4% (w/v) paraformaldehyde overnight. Then, the fixed tissue specimens were embedded into paraffin for sectioning, or in Tissue-Tek O.C.T. Compound (#4583; Sakura Finetek, Tokyo, Japan) after serial penetration with 5–25% (w/v) sucrose-PBS for frozen sectioning.

The samples were processed for immunohistochemistry for each assessment. Four images were captured per ATM sample by microscopy (BZ-X710 All-in-One Fluorescence Microscope; Keyence, Osaka, Japan). Histological assessments of the samples were performed by the software cellSens (Olympus, Tokyo, Japan).

#### Immunohistochemistry of citrullinated histone H3

Frozen sections (7 μm) were permeabilized with PBS containing 0.1% (w/v) sodium citrate and 0.1% (v/v) Triton X-100 (Sigma-Aldrich) at 4°C for 10 min. This was followed by washing with PBS and subsequent blocking with PBS containing 0.5% (v/v) BSA and 5% (v/v) goat serum at RT for 90 min. The sections were reacted with primary anti-histone H3 (citrulline R2+R8+R17) antibody-ChIP Grade (#ab5103; Abcam) (1:500) diluted with PBS containing 0.3% (v/v) BSA and 1% (v/v) goat serum at 4°C overnight. The sections were also incubated with Iisotype control antibodies under the same procedure. The reacted specimens were washed with PBS and then incubated with goat anti-rabbit IgG (H+L) highly cross-adsorbed secondary antibody, Alexa Fluor 488 (#A-11034; Invitrogen) (1:200) diluted with PBS containing 0.3% (v/v) BSA and 1% (v/v) goat serum at RT for 90 min. The samples were washed with PBS and mounted in 4′,6-diamidino-2-phelylindole (DAPI)/1,4-diazabicyclo [2.2.2] octane (DABCO).

#### Fgn immunohistochemistry

After their deparaffinization of the tissue sections (2.5 μm), the antigens from the 2.5 μm sections were was retrieved by boiling with Target Retrieval Solution (#S1699, DAKO, Glostrup, Denmark) at 98°C for 20 min, followed by cooling down at RT. The sections were blocked with Protein Block Serum-Free (#X0909; DAKO) at RT for 30 min and incubated with rabbit polyclonal anti-mouse Fgn antibody (#ab27913; Abcam) (1:200) at 4°C overnight. After incubation, intrinsic peroxidase in the tissue sections was blocked with 3% (v/v) H_2_O_2_/methanol at RT for 10 min. Subsequently, the sections were incubated with secondary anti-rabbit HRP antibody at RT for 60 min and then reacted with 3,3′-diaminobenzidine solution for 90 s. Finally, the sections were stained with hematoxylin and mounted with Malinol 750cps (#20091; Muto Pure Chemicals Co., Ltd., Tokyo, Japan). Isotype control antibodies were also processed by the same procedure.

#### Evaluation of angiogenesis

To stain capillaries, 7 μm frozen sections (after blocking) were incubated with rat anti-mouse CD31 antibody (#550274, BD Biosciences) diluted (1:500) with PBS containing 0.1% (v/v) BSA and 5% (v/v) goat serum at 4°C overnight. Isotype control antibodies Purified rat IgG (#6-001-A; R&D systems, Minneapolis, MN, USA) as a control antibody was also incubated in the same manner. The reacted sections were incubated with the goat anti-rat IgG (H+L) cross-absorbed secondary antibody, Alexa Fluor 594 (#A-11007; Invitrogen) (1:200) diluted with PBS containing 0.3% (v/v) BSA and 1% (v/v) goat serum at RT for 60 min. After the sections were washed with PBS, they were mounted with DABCO.

#### Evaluation of arteriogenesis

Frozen sections (7 μm) were blocked using the avidin/biotin blocking kit (#SP-2001; Vector Labs, Burlingame, CA, USA) for 15 min and then incubated with Isolectin GS-IB4 from *Griffonia simplicifolia* and biotin–XX conjugate (#121414; Molecular Probes) (1:100) diluted with 1 mM Ca^2+^, Mg^2+^ PBS/0.2% (v/v) Triton X-100 (Sigma-Aldrich) at 4°C overnight. The tissue sections were washed with PBS and then incubated with PBS containing streptavidin, Alexa Fluor 488 conjugate (#S11223; Invitrogen) (1:500), and the anti-α-smooth muscle actin (αSMA)- Cy3 antibody (#C6198; Sigma-Aldrich) (1:200) at RT for 60 min. Next, the sections were washed with PBS and mounted with DABCO.

### Statistical analysis

Using SPSS version 25 (IBM, Armonk, New York, USA) or Prism 6.0 (GraphPad Software, San Diego, USA), one-way ANOVA with Bonferroni’s correction as a *post hoc* comparison test was used to test whether differences among the three groups were significant. Comparisons of the values between two groups were analyzed using the Mann–Whitney *U-*test. Differences were considered significant at P < 0.05. Data values are indicated as mean ± standard deviation.

## Results

### Batroxobin inhibited human NETs under inflammatory conditions *in vitro*

**Fig 1:**
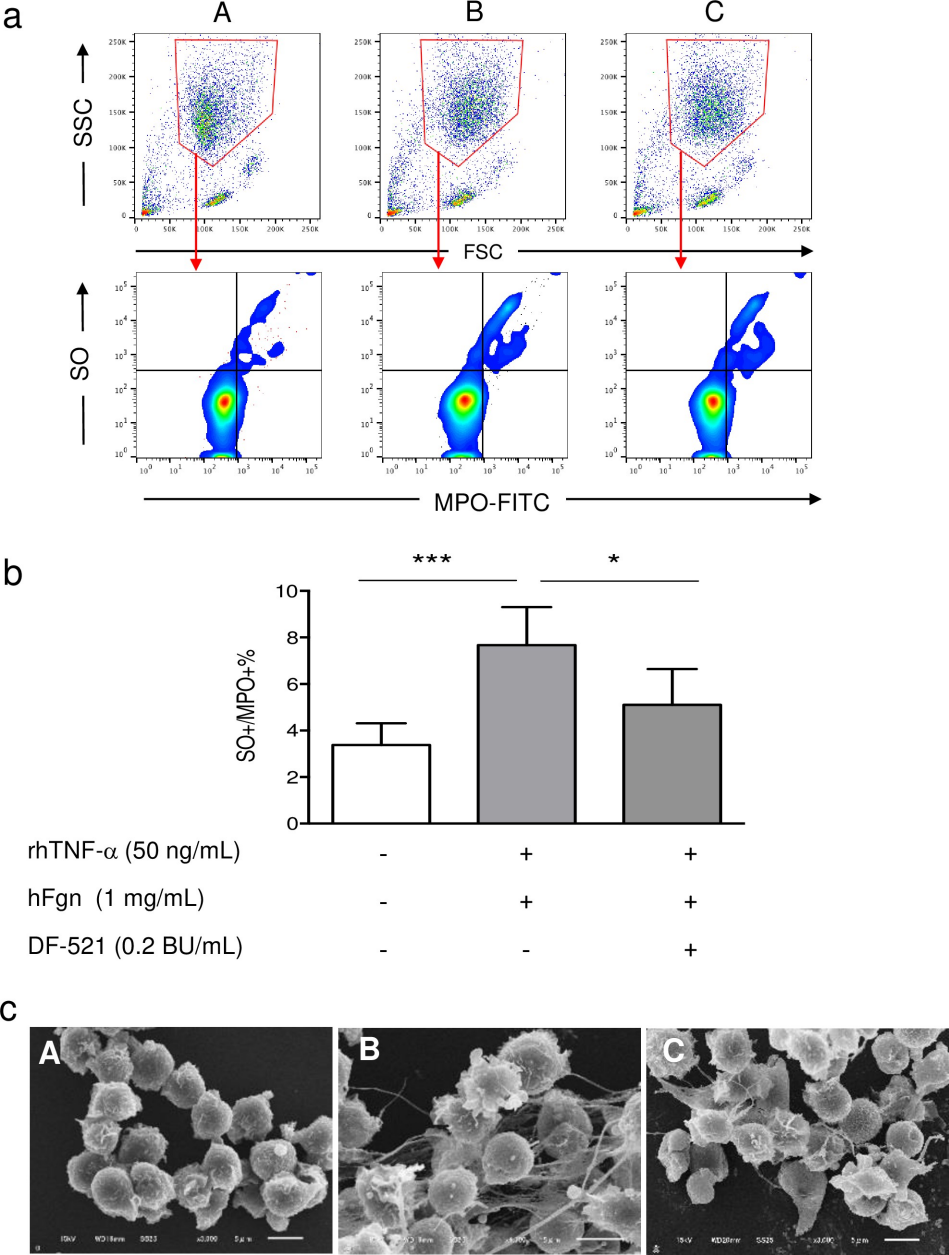
The inhibitory effect of batroxobin on human NETs in the presence of TNF-α with human Fgn. (a) Upper panels show flow cytometry scattergrams of isolated cells stained with SO and anti-MPO antibody-FITC in each condition. Lower panels show scattergrams of the population stained with anti-MPO antibody–FITC gated from the respective panels in each condition. (b) The bar graphs indicate the percentages of NETs doubly stained with SO and anti-MPO antibody-FITC as mean ± SD. ***P < 0.001, *P < 0.05, n = 3. (c) SEM images show the features of NETs under the respective conditions. Scale bar = 5 μm. (A), depleted rhTNF-α and hFgn; (B) or (C), absence or presence of rhTNF-α with hFgn. rhTNF-α = ng/mL, hFgn = 1 mg/mL.

In **Fig 1A and 1B**, NETs were induced in the presence of rhTNF-α and hFgn (B) to mimic an inflammatory environment when compared to the absence of both agents (A); SO-positive and MPO-positive (SO^+^MPO^+^) neutrophils gated with red lines increased significantly (P = 0.001), from 3.4% to 7.7%. Batroxobin inhibited this increase to 5.1% (P = 0.04) (C), indicating a 0.66-fold inhibitory effect by (B). The results corresponded with SEM images **(Fig 1C)**. In image B, NETs are visible as fibrous structures in extracellular spaces but not in image C or image A; the structures are shrunken in image C.

### Batroxobin inhibited NETs with Fgn deposition and subsequent tissue damage in the ATM of ischemic hindlimbs

**Fig 2:**
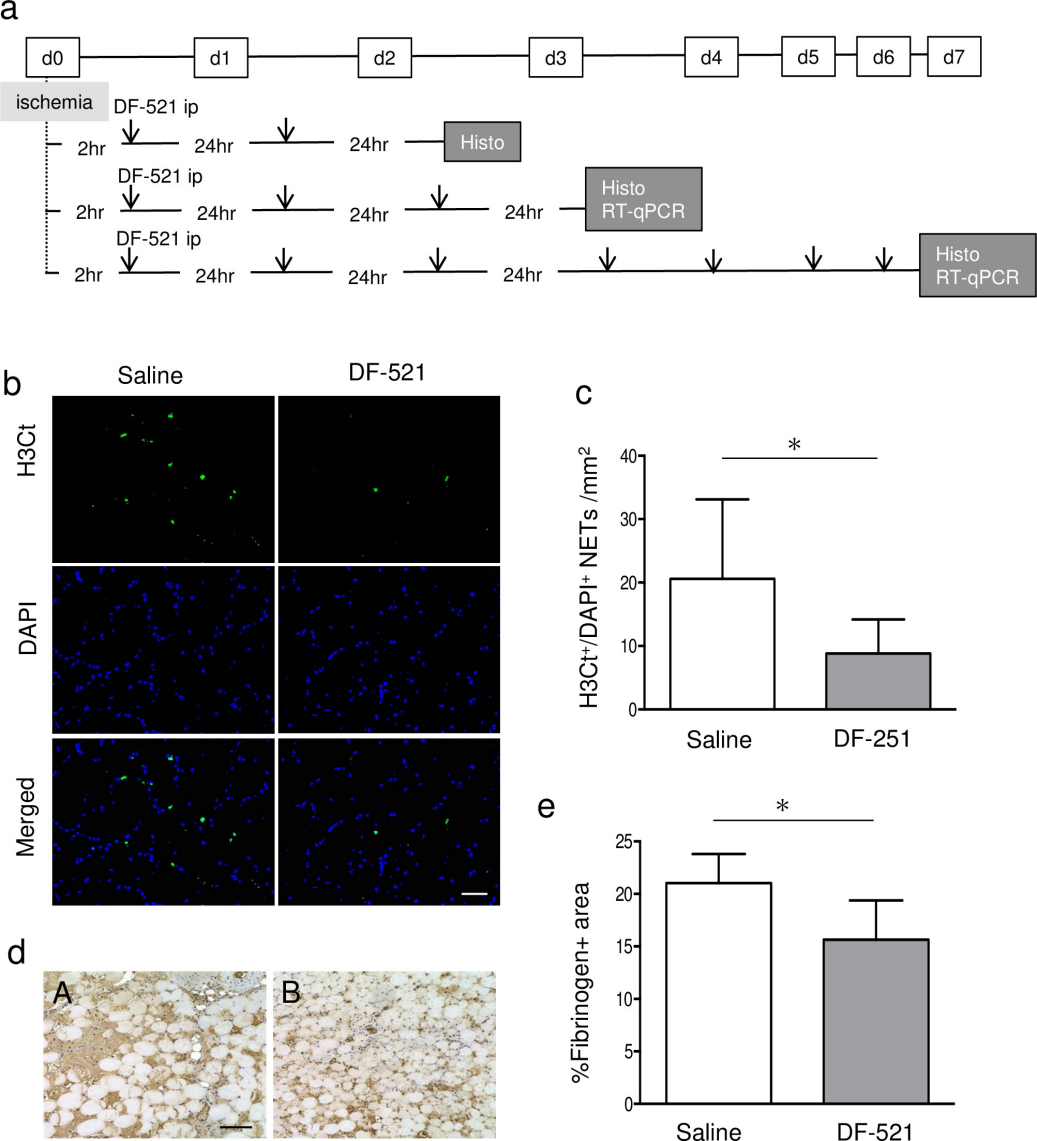
Evidence of the anti-inflammatory effects of batroxobin in ischemic ATM. (a) The protocol of the animal experiment. (b, c) NETs detected by staining with anti-histone H3 (citrulline R2+R8+R17) antibody and DAPI in ischemic tissues on day 2 post ischemia. (b) The representative pictures of H3Ct^+^/DAPI^+^ NETs in ischemic ATMs. Scale bar = 50 μm. (c) The bar graph shows the number of H3Ct^+^/DAPI^+^ NETs per square millimeter as mean ± SD. *P < 0.05, n = 6. (d, e) The deposited fibrinogen in the tissue of ischemic ATMs on day 2 post ischemia. (d) The representative pictures of tissue-recruited Fgn in ischemic ATMs. The brown area indicates tissue-recruited Fgn. Scale bar = 100 μm. (A) saline control, (B) DF-521-administered group. Scale bar = 100 μm. (e) The bar graph shows the percentage of Fgn-occupied area in ischemic tissue as mean ± SD. *P < 0.05, n = 6.

As indicated in the experimental protocol scheme of **Fig 2A**, a couple of immunohistochemistry analyses were performed. On day 2 post ischemia, the number of citrullinated histone H3^+^ (H3Ct^+^)/DAPI^+^ cells per square millimeter of the tissue cross section, indicating NETotic cells in the ATMs of DF-521-treated mice, decreased compared to the control (8.8 ± 5.4/mm^2^ for DF-521 vs. 20.6 ± 12.5/mm^2^ for the control; P = 0.01) **(Fig 2B and 2C)**. Furthermore, batroxobin reduced the area of Fgn deposition in ischemic ATMs (16% in DF-521 vs. 21% in control; P = 0.01) **(Fig 2D and 2E)**. The findings indicate that batroxobin as a dysfibrinogenic agent not only decreases Fgn deposition but also protects against NETs in ischemic tissue to alleviate acute inflammation.

#### Gene expression profiles demonstrated the effect of batroxobin in accelerating ischemic tissue repair

**Fig 3:**
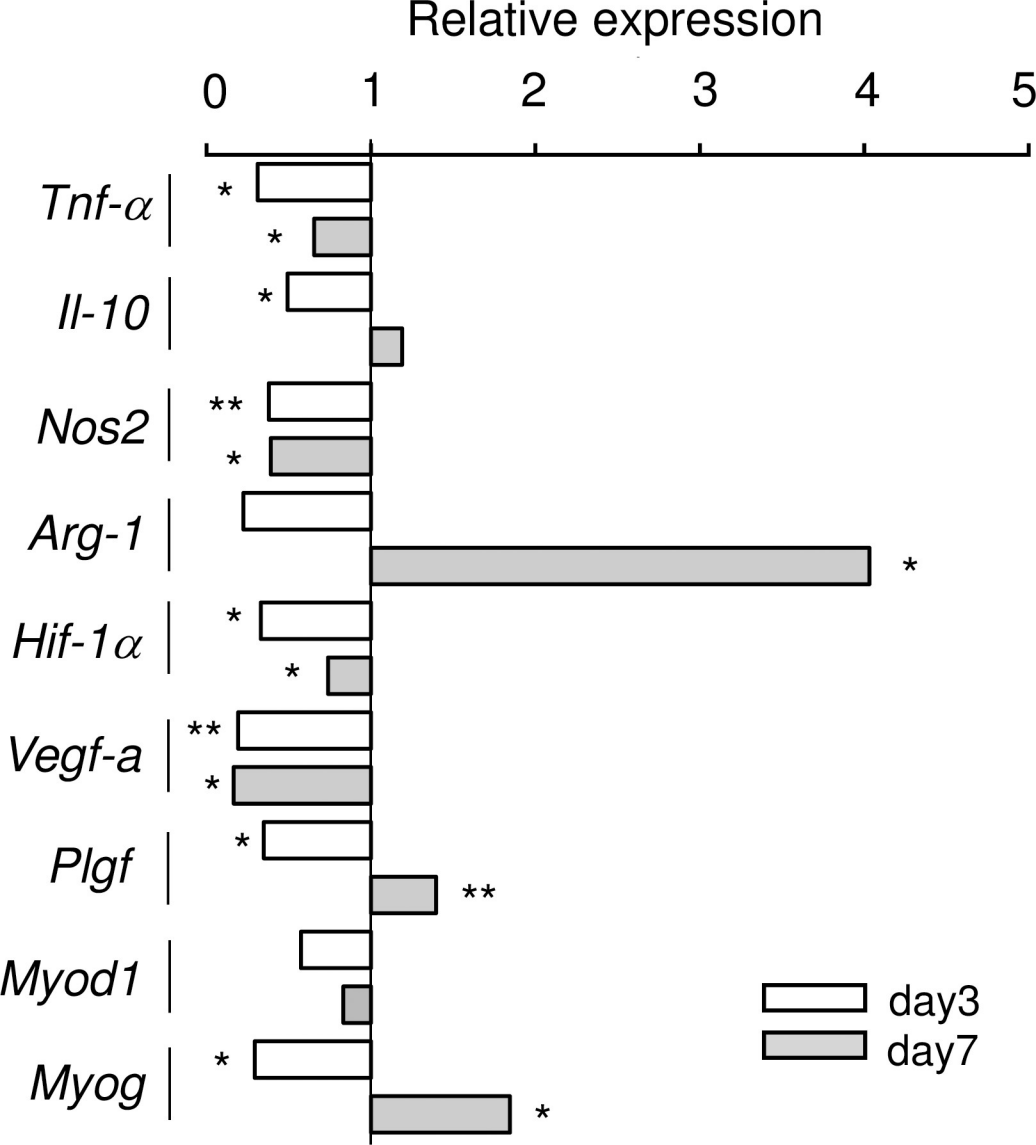
The gene expression profiles of ischemic ATMs. The bar graph shows the fold change of expression of each gene in ischemic ATMs in DF-521-administered mice vs. saline control mice as mean. Clear bar = day 3, gray bar = day 7, **P < 0.01, *P < 0.05, n = 5.

In the present experiments, the acute inflammation in ischemic ATMs was alleviated in the DF-521-treated group. The level of *Tnf-α* expression was significantly lower (0.31-fold) in the control on day 3, and it remained 0.66-fold less even on day 7. Furthermore, the expression of nitric oxide synthase2 (*Nos2*) (inducible), an inflammatory M1 macrophage marker, remained 0.38–0.39-fold lower than that of the control from day 3 to day 7. The expression of interleukin-10 (*Il-10*) was suppressed 0.49-fold in the DF-521-treated group versus the control on day 3, whereas it was subsequently upregulated 1.2-fold on day 7 (albeit without statistical significance). Of note, the expression level of arginase-1 (*Arg-1*), a marker of the anti-inflammatory M2 macrophage, was 0.22-fold of the control, similar to the other inflammation-related markers, but conversely, it was 4.0-fold higher than that of the control on day 7. These results indicate that batroxobin alleviated acute inflammation in ischemic ATM (**Fig 3**).

With regard to inflammatory angiogenesis, hypoxia inducible factor-1α (*Hif-1α*) gene expression was 0.33- and 0.74-fold lower than that of the control on both days. Similarly, vascular endothelial growth factor-a (*Vegf-a*) gene expression induced by Hif-1α significantly remained 0.19- or 0.17-fold lower than that of the control. As for the gene expression inducing functional angiogenesis with arteriogenesis, placental growth factor (*Plgf*) expression stayed 0.35-fold lower than that of the control on day 3, increasing 1.4-fold in the DF-521-treated group on day 7. These results demonstrate that the mitigation of the inflammatory microenvironment by batroxobin in ischemic ATM suppressed inflammatory angiogenesis at the acute phase, subsequently accelerating functional angiogenesis with arteriogenesis for vascular regeneration (**Fig 3**).

With regard to myogenesis, the expression of *Myod1*, a skeletal muscle transcriptional factor determining skeletal muscle cell fate and myoblast proliferation, was maintained at 0.58- and 0.83-fold lower than that of the control on both days. In addition, the expression level of myogenin (*Myog*), a myoblast differentiation factor at late stages of myogenesis, stayed 0.29-fold lower on day 3, increasing to 1.9-fold on day 7 (**Fig 3**). Together, the findings indicate that batroxobin contributed to restoring skeletal muscle injury to its intact state but not so much to inducing cell fate determination of satellite cells or myoblast proliferation in myogenesis. The values of each gene expression ratio are indicated in **S3 Table**.

### Batroxobin accelerated tissue repair in ischemic hindlimbs

#### Revascularization in angiogenesis and arteriogenesis

**Fig 4:**
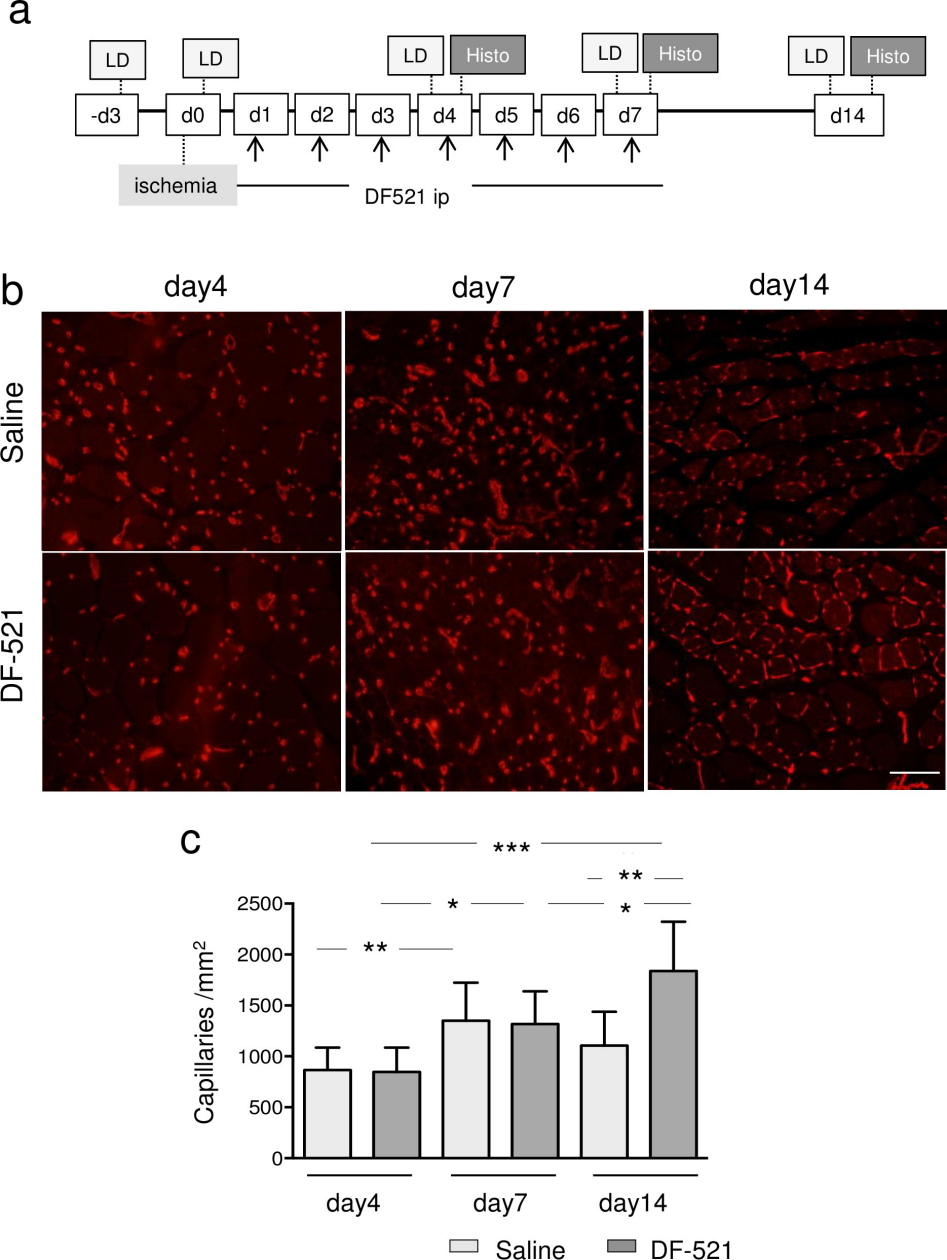
Sequential features of angiogenesis in ischemic ATMs of each group. (a) The protocol of the experiment. (b) The representative pictures of angiogenesis on day 4, day 7, and day 14 post ischemia. Angiogenesis was evaluated by fluorescence immunohistochemistry with the Alexa-594-conjugated anti-mouse CD31 antibody. Scale bar = 100 μm. (c) The bar graph shows the capillary density (capillaries /mm^2^) at each time point as mean ± SD. Clear column = control mice, gray column = DF-521-administered mice. *** *P* < 0.001, **P < 0.001, *P < 0.05, n = 6.

**Fig 5:**
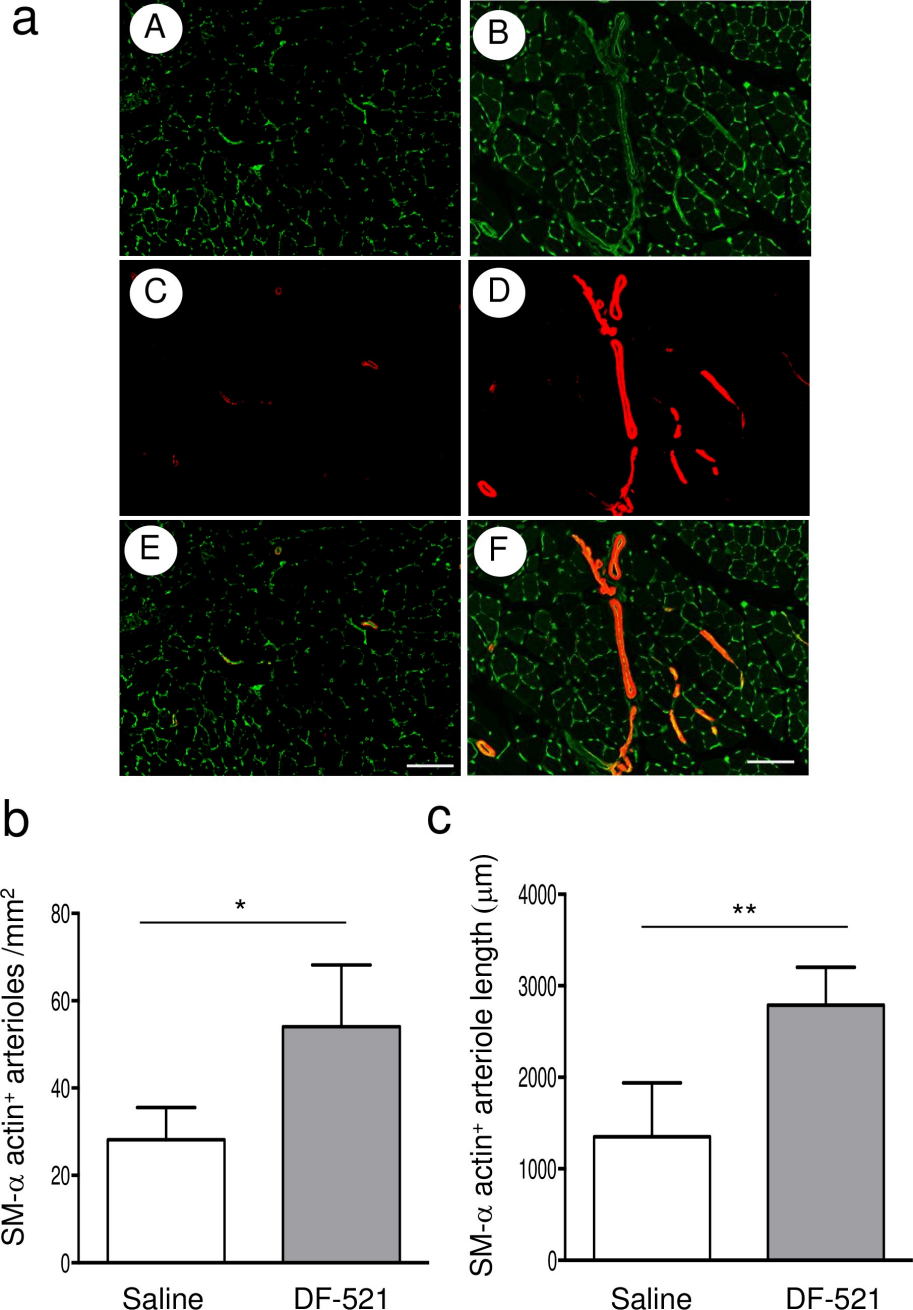
Arteriogenesis in ischemic ATMs of each group. (a) The representative pictures of arteriogenesis in ischemic ATMs on day 14. Arteriogenesis was evaluated by fluorescence immunohistochemistry with the anti-αSMA antibody-Cy3 conjugate and isolectin B4-Alexa488 conjugate. (A, C, E) control group, (B, D, F) DF-521-administered group. (A, B) Isolectin B4-Alexa488 conjugate-stained images, (C, D) anti-αSMA antibody-Cy3 conjugate-stained images, (E, F) merged images. Scale bar = 200 μm. (b, c) The bar graphs indicate the number and total length of doubly stained arterioles as mean ± SD. **P < 0.001, *P < 0.05, n = 6.

The capillary density of ischemic ATM sequentially and significantly increased in the DF-521-administered group from day 4 to day 14 post ischemia (848 ± 238/mm^2^ on day 4, 1,318 ± 321/mm^2^ on day 7; P = 0.04 vs. day 4, 1,839 ± 483/mm^2^ on day 14; P = 0.036 vs. day 4 or day 7). On day 14, the capillary density of DF-521-administered mice increased compared to the control (1,106 ± 332/mm^2^ in the control; P = 0.0019). In contrast, the capillary density of ischemic ATM in the control remained low on day 14, although it transiently increased on day 7 (1,352 ± 372/mm^2^ on day 7 vs. 865 ± 222 on day 4; P = 0.014) **(Fig 4A–4C)**. Arteriogenesis was evaluated by measuring the number and length of αSMA-positive arterioles per square millimeter of the tissue cross section. The number of arterioles was significantly higher in the DF-521-treated group than in the control (54 ± 14.1/mm^2^ in DF-521 vs. 28 ± 7.4/mm^2^ in the control; P = 0.032) **(Fig 5A and 5B)**. Furthermore, the total length in the treatment group increased (2,790 ± 412/mm^2^ in DF-521 vs. 1,352 ± 585/mm^2^ in the control; P = 0.008) **(Fig 5A–5C)**.

#### Blood perfusion recovery

**Fig 6:**
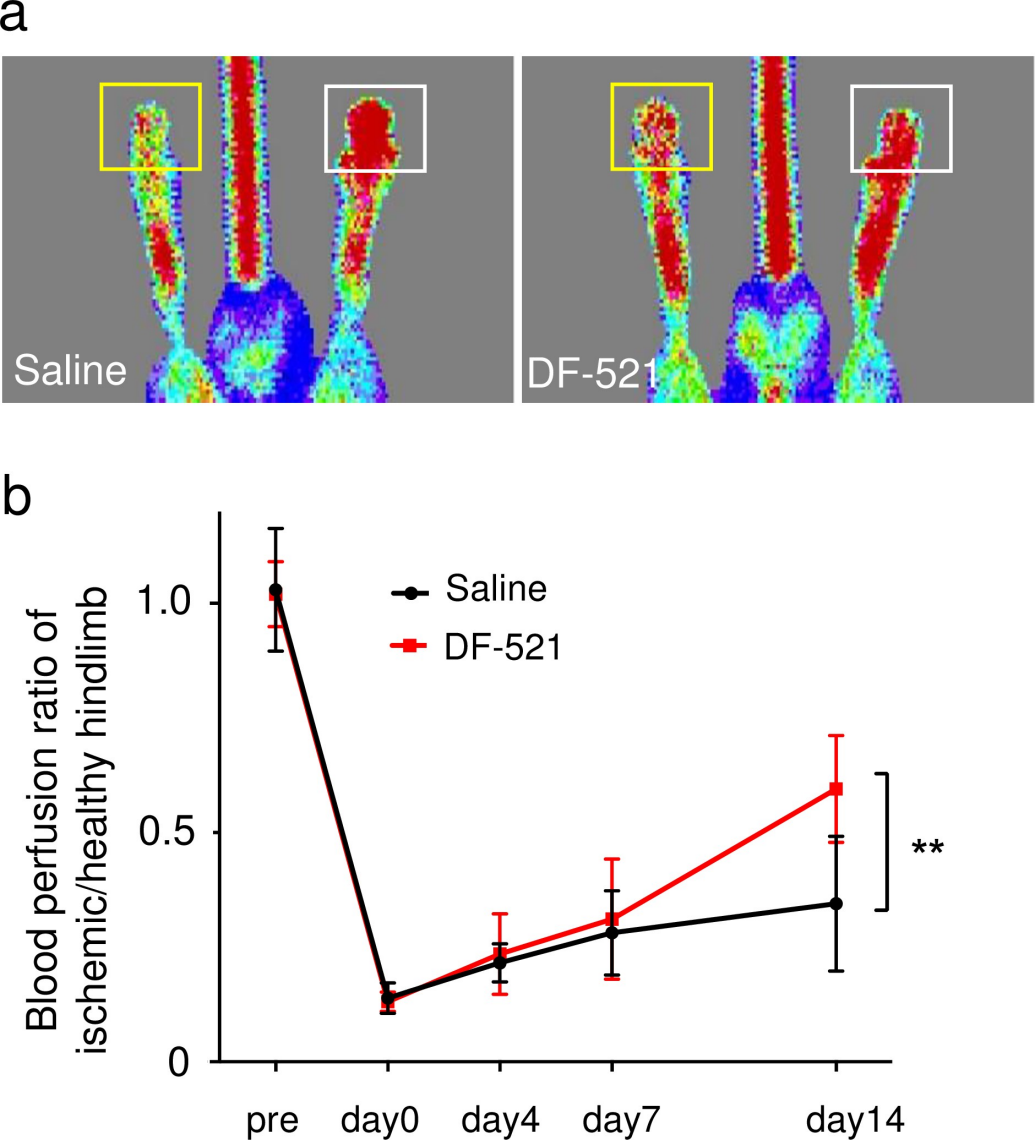
Blood perfusion analysis by laser Doppler imaging in DF-521-administered mice vs. control mice. (a) The representative pictures of laser Doppler imaging in each mouse. The yellow or white square shows the region of interest to measure flux intensity (arbitrary unit) indicating blood perfusion in the ischemic or healthy toe. (b) The line graph shows the blood perfusion ratio of the ischemic hindlimb vs. healthy hindlimb in each group as mean ± SD. *P < 0.05, n = 10.

The blood perfusion of ischemic hindlimbs in the mice administered DF-521 daily for 7 days did not differ from that of the control. On day 14 post ischemia, it significantly improved versus the control; the ratio of the ischemic to the contralateral hindlimb was 0.60 ± 0.12 in DF-521 vs. 0.35 ± 0.15 in the control (P = 0.0029, n = 10) **(Fig 6A and 6B)**.

#### Skeletal muscle maturation

**Fig 7:**
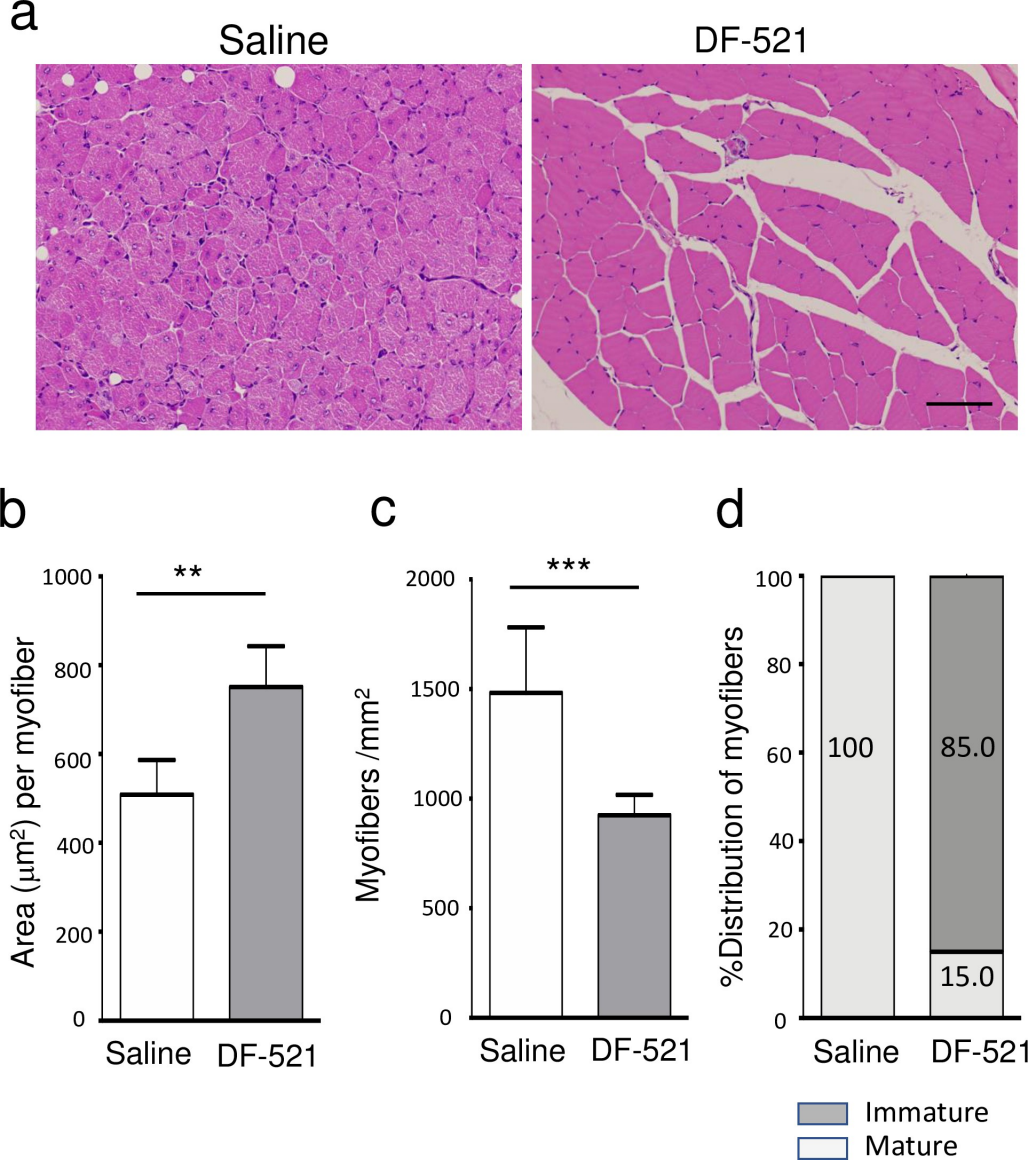
Histological features of ATMs in ischemic hindlimbs. (a) The images are the features of myofibers in ischemic ATMs on day 14 post ischemia. Scale bar = 200 μm. The bar graphs show the area per myofiber (μm^2^) (b), the number of myofibers (c) and the distribution of immature and mature myofibers (%) (d) per square millimeter in the tissue cross section as mean ± SD. **P < 0.01, ***P < 0.001, n = 7.

Hematoxylin staining revealed that batroxobin advanced skeletal muscle maturation in the injured tissue of ischemic ATM **(Fig 7, S3 Fig)**. On day 14, thin myofibers with central nuclei (immature myofibers) were belatedly induced in the control, while myofibers almost matured, as abundantly detected by the thick cellular bodies with peripheral nuclei in the DF-521 group **(Fig 7A)**. The average area per myofiber in a tissue cross section of ischemic ATM were significantly thicker in the DF-521-treated group than in the control (751 ± 91.2 μm^2^ in DF-521 vs. 509 ± 77.9 μm^2^ in the control; P = 0.0012) **(Fig 7B)**. The number of myofibers per square millimeter of the tissue cross section was inversely lower in the DF-521-treated group (1,483 ± 298.8/mm^2^ in DF-521 vs. 924 ± 94.0/mm^2^ in the control; P = 0.0006) **(Fig 7C)**. Further, in the percent distribution of immature and mature myofibers per square millimeter, immature myofibers occupied 100% in the control, while mature myofibers occupied 85% in the DF-521-treated group **(Fig 7D)**.

## Discussion

Batroxobin, a defibrinogenating agent from snake venom, has been reported to suppress inflammation and circumvent hyperviscosity. However, the anti-inflammatory function of batroxobin has yet to be discussed from the point of view of NETs inducing tissue injury and providing a microthrombogenic platform for disturbing circulation.

In the present study, using a mouse hindlimb ischemia model, we demonstrated that batroxobin abrogated NETs involved in tissue injury, along with defibrinogenation at the acute ischemic phase, thereby expediting ischemic tissue repair while promoting revascularization and skeletal muscle maturation.

### Inhibitory effect of batroxobin on NETs triggering inflammation and ischemic tissue injury

In our *in vitro* human NET assay induced by TNF-α in the presence of Fgn, batroxobin interfered with NET formation **(Fig 1)**. In a previous *in vivo* study, batroxobin decreased C3d or C9 deposition in the alternative complement pathway (AP), as well as intercellular adhesion molecule-1 in intracranial hemorrhagic sites in rats [15]. This suggests that one of the anti-inflammatory functions of batroxobin is exerted through the inactivation of the AP. Regarding this, neutrophils themselves carry critical components of the AP, such as C3, properdin, complement factor P, or complement factor B. In other words, NETs *per se* can activate the AP [30].

Conversely, activated complements may also induce NET formation [31]. In brief, NETs can serve as a positive feedback platform between NET formation and the AP. As shown in **Fig 2B and 2C**, immunohistochemical analysis revealed that batroxobin hampered NETs in the ischemic ATM. At this point, whether batroxobin may primarily disturb NET formation, the AP, or both remains to be elucidated, but our finding shows that batroxobin exerted the anti-inflammatory effect by suppressing NET formation as a scaffold of the AP. All complement pathways containing the AP are well-known for leading a common terminal pathway for forming a cytolytic membrane attack complex, which causes tissue injury by its cytocidal ability [32].

With regard to the relationship between batroxobin and tissue Fgn, Fgn deposition in ischemic tissue decreased in batroxobin-treated mice **(Fig 2D and 2E)**, which might reflect its defibrinogenating effect. Fgn is known to enhance inflammation and to be an early inflammatory biomarker [33–35]. More recently, Fgn was reported to induce the polarization of mouse bone marrow macrophages to M1 macrophages expressing TNF-α [36].

Herein, the decreased Fgn deposition in batroxobin-treated mice on day 2 of the early ischemic phase **(Fig 2D and 2E**) showed that batroxobin reduced ischemic inflammation via the defibrinogenating effect. Interestingly, during the same acute phase of day 2, batroxobin inhibited NETs, as mentioned earlier. On this aspect, Healy et al. recently reported that Fgn colocalizes with the extracellular DNA of NETs, as well as the cell body of neutrophils, while the other coagulation factors-prothrombin, factor X, and factor VIIa-bind only to the cell body [25]. In several recent reports [27,37–39], NETs have been shown to serve as platforms for not only microthrombogenesis by such coagulation factors but also inflammation by the AP.

In microvasculatures in particular, NETs cause endothelial cell death [40], thereby enhancing microvascular inflammation. Even in ischemic diseases, Fgn associated with NETs is considered to play an important role in “thromboinflammation,” which has recently been described in antineutrophil cytoplasmic antibody-associated vasculitis [23]. Therefore, batroxobin conceivably protected against the thromboinflammation caused by hindlimb ischemia in the present study, although the molecular mechanism underlying the inhibitory effect of batroxobin on NETs remains to be ascertained.

After alleviating thromboinflammation in the acute phase of ischemia, batroxobin accelerated the tissue repair involved in vascular and skeletal muscle regeneration in ischemic ATM. To understand the association between the alleviated thromboinflammation and the accelerated tissue repair, we investigated the kinetics of transcripts in the injured tissue that play important roles in pro- or anti-inflammation, together with vascular and skeletal muscle regeneration **(Fig 3)**.

#### The TNF-α/IL-10 axis benefits anti-inflammation

TNF-α and IL-10 are known to be pro- or anti-inflammatory cytokines in ischemic diseases [41,42] and its animal models [43,44]. In this study, murine *Tnf-α* expression in injured tissue from day 3 to day 7 of the acute to the subacute phase was sustained at lower levels by batroxobin injection, while *Il-10* expression was similarly lower in the acute phase but was inversely upregulated in the subacute phase. This finding of the TNF-α/IL-10 axis corresponds with the inhibition of batroxobin in the preceding NET activation in ischemic events.

#### The NOS2/ARG-1 axis is involved in tissue repair

The NOS2/ARG-1 axis features a pattern similar to that of the TNF-α/IL-10 axis. *Nos2* expression in the acute to subacute phases remained lower by batroxobin injection, while *Arg-1* expression in the injured tissue was markedly upregulated in the subacute phase. NOS2 and ARG-1 share l-arginine as the substrate in macrophages of myeloid cells [45,46]. NOS2 mainly metabolizes l-arginine to NO, or alternatively to superoxide (O_2_^-^) under inadequate l-arginine supply. Both NO and O_2_^-^ finally react and produce cytotoxic peroxynitrate (ONOO^-^) to induce tissue damage and enhance inflammation. However, ARG-1 metabolizes the substrate to urea and l-ornithine; the latter is subsequently metabolized to l-proline and putrescine, which promote tissue repair and cell proliferation. The local activation of ARG-1 has been reported to be advantageous to wound healing [47] or injured retinal neurovascular repair [48]. Considering the similar feature of the TNF-α/Il-10 axis, batroxobin suppresses the production of the pro-inflammatory factors TNF-α or NOS2 to a lesser extent, while preferentially promoting the anti-inflammatory factors IL-10 or ARG-1 in the subacute phase.

### Expediting vascular regeneration of angiogenesis and arteriogenesis

On day 14 of the chronic phase, batroxobin augmented capillary formation in the ischemic tissue but not in the control **(Fig 4B and 4C)**. Furthermore, pericyte-recruited vasculatures increased with elongation compared to the control **(Fig 5A–5C)**. Correspondingly, blood perfusion in the ischemic hindlimb was preferentially restored **(Fig 6)**. These findings show that batroxobin promoted the vascular regeneration of angiogenesis and arteriogenesis following the alleviation of inflammatory tissue injury. As a longstanding concept, the smooth or appropriate activation of the so-called HIF-1α/VEGF-A/PLGF axis is required for vascular regeneration [49,50]. I n this study, the essential role of PLGF without the activation of the HIF-1α/VEGF axis was unexpectedly discovered, as described below.

Batroxobin suppressed the gene expression of *Hif-1α* and *Vegf-a* in ischemic tissue during both phases **(Fig 3)**. Considering the decline in NETs providing a microthrombogenic platform in the acute phase, batroxobin might exacerbate microcirculation to a lesser extent, thereby escaping more severe hypoxia. Thus, the lower level of *Hif-1α* transcripts conceivably led to the downregulation of *Vegf-a* gene expression, as *Hif-1α* is a potent transcriptional factor of *Vegf-a* mRNA [51,52].

As a consequence, the reduction in the gene expression of *Hif-1α* and *Vegf-a* in ischemic tissue implies that batroxobin-induced NET inhibition alleviates thromboinflammation, thereby resulting in some improvement in microcirculation.

Next, *Plgf* expression was lower on day 3 of the acute phase, increasing on day 7 of the subacute phase, which differs from *Hif-1α* and *Vegf-a*. PLGF, a VEGF-A homolog sharing its VEGF receptor-1 (fms like tyrosine kinase-1 [FLT-1]), is an angiogenic and arteriogenic growth factor [53–55]. Functionally, unlike VEGF, PLGF contributes to angiogenesis and arteriogenesis with less permeability, characterized as physiologically more long-lasting vascular formation [49], and its expression in M2 macrophages has been reported to promote angiogenesis [56]. Herein, *Plgf* expression on day 7 of the subacute phase was probably upregulated in M2 macrophages in the ischemic tissue. More PLGF secretion from M2 macrophages is considered to recruit more abundant pericytes/smooth muscle cells expressing FLT-1 [57–59], thereby promoting arteriogenesis. Concomitantly, the upregulated *Arg-1* gene expression *per se* in the tissue on day 7 might have contributed to arteriogenesis and M2 macrophage accumulation around the perivascular region [60].

### Accelerating skeletal muscle regeneration

Batroxobin-treated mice exhibited accelerated myofiber maturation in ischemic ATM on day 14 **(Fig 7)**; in the control, immature myofibers with centrally located nuclei still remained, whereas their maturation to myofibers with eccentrically located nuclei was almost completed.

Considering the above, batroxobin might protect inflammatory NETs from inducing acute tissue injury, thereby showing earlier restoration, as well as lesser tissue damage.

MyoD1 is a transcriptional factor that induces the proliferation of satellite cells and myoblasts, i.e., it makes MYOG differentiate myoblasts into myotubes or myofibers [61,62]. The gene expression of *Myod1* was somewhat lower in the treated mice than that of the control, although not significantly, while *Myog* expression was considerably lower on day 3 but inversely upregulated-that too, dominantly-on day 7. *Myod1* expression might be due to the reduced need for considerable proliferation of satellite cells or myoblasts, owing to the preceding alleviated tissue injury.

The tissue injury in batroxobin-treated mice might have caused more satellite cells to escape from cell death. Consequently, the upregulation of *Myog* expression might have occurred because of the predominant maturation through myoblasts to myofibers rather than by the proliferation of the remaining satellite cells for earlier muscle regeneration.

### Limitations of this study

This study demonstrated how batroxobin accelerates tissue repair from the acute ischemic phase to the subacute phase. However, some missing links still remain to be unveiled, especially in terms of basic science, such as whether batroxobin directly interferes with NET formation even in the absence of Fgn or indirectly through its defibrinogenating effect only in the presence of Fgn. Surely, in the latter case, any effect of Fgn on NET formation has to be verified first. However, given that Fgn colocalizes with coagulation factors in NETs, this is adequately anticipated [25]. Furthermore, the molecular mechanism of NET inhibition via batroxobin remains to be elucidated in future studies.

### Clinical perspectives for batroxobin

The reclamation of novel clinical applications in previously developed medicines is an emerging field today. The anti-inflammatory effect of batroxobin through the inhibition of neutrophil activation is expected to expand the clinical applications for various inflammatory diseases, particularly in terms of drug repositioning. Clinically applicable medicines inhibiting NETs have recently been under development, such as protein arginine deiminase 4 inhibitors, signal inhibitory receptor on leukocytes, and DNase I [63]. In the clinical setting, batroxobin is expected be an important agent in cutting-edge drug repositioning for targeting NETs from its current use solely for microcirculation disorders.

## Conclusion

Batroxobin suppresses microthromboinflammation by inhibiting NETs and by defibrinogenation, thereby accelerating ischemic tissue repair. Such a regulatory approach against microthromboinflammation provides a new therapeutic approach for applications in regenerative and inflammatory medicine. Above all, batroxobin might be valuable as an already clinically applied medicine against diverse NET-induced inflammatory diseases.

## Supporting information

S1 Table The list of abbreviations. (DOCX)

S2 Table The lists of reagents and items used in the study. (DOCX)

S3 Table Relative gene expression in ischemic ATM of the DF-521-administered group vs. the control (Fig 3). (DOCX)

S1 Fig Flow cytometric features of the neutrophils isolated from human peripheral blood. (TIF)

S2 Fig Ligated portions of the femoral artery for creating an ischemic hindlimb. (TIF)

S3 Fig Histological features of ATMs of the other mice in ischemic hindlimbs. (TIF)
