# Supplementary material for: Batroxobin accelerated tissue repair via neutrophil extracellular trap regulation and defibrinogenation in a murine ischemic hindlimb model
Source: PLoS One. 2019 Aug 16;14(8):e0220898. doi: 10.1371/journal.pone.0220898 (PMC6697371; doi:10.1371/journal.pone.0220898)
Supplement: S1 Table — (DOCX) [file pone.0220898.s001.docx]

**S1 Table. The list of abbreviations.**

| **Complete name** | **Abbreviation** |
| --- | --- |
| fibrinogen | Fgn |
| neutrophil extracellular traps | NETs |
| myeloperoxidase | MPO |
| phosphate-buffered saline | PBS |
| room temperature | RT |
| ethylenediaminetetraacetic acid | EDTA |
| fetal bovine serum | FBS |
| tumor necrosis factor-α | TNF-α (*Tnf-α*) |
| Sytox Orange | SO |
| scanning electron microscopy | SEM |
| anterior tibial muscle | ATM |
| 4’,6-diamidino-2-phelylindole | DAPI |
| 1,4-diazabicyclo [2.2.2] octane | DABCO |
| α-smooth muscle actin | αSMA |
| citrullinated histone H3 | H3Ct |
| nitric oxide synthase2 | NOS2 (*Nos2*) |
| interleukin-10 | Il-10 (*Il-10*) |
| arginase-1 | ARG-1 (*Arg-1*) |
| hypoxia-inducible factor-1α | HIF-1α (*Hif-1α*) |
| vascular endothelial growth factor-a | VEGF-A (*Vegf-a*) |
| placental growth factor | PLGF (*Plgf* ) |
| myogenin | MYOG (*Myog*) |
| fms-like tyrosine kinase-1 | FLT-1 |

The abbreviations with the complete names are listed in the sequential order in which they appear in the text. The italic letters in the round brackets show the abbreviations of the genes corresponding to the factors listed in the left column.
