# Supplementary material for: Batroxobin accelerated tissue repair via neutrophil extracellular trap regulation and defibrinogenation in a murine ischemic hindlimb model
Source: PLoS One. 2019 Aug 16;14(8):e0220898. doi: 10.1371/journal.pone.0220898 (PMC6697371; doi:10.1371/journal.pone.0220898)
Supplement: S2 Table — (DOCX) [file pone.0220898.s002.docx]

**S2 Tables. The lists of reagents and items used in the study.**

**Items used for neutrophil isolation and NET induction**

| **Items** | **Catalog No.** | **Company** |
| --- | --- | --- |
| PolymorphoPrep density gradient media | #1114683 | PROGEN Biotechnik GmbH |
| BD Pharm Lyse lysing buffer | #555899 | BD Biosciences |
| RPMI-1640 | #187-02021 | FUJIFIM Wako Pure Chemical |
| Penicillin–streptomycin | #P4333 | Sigma-Aldrich |
| Human fibrinogen | #F4883 | Sigma-Aldrich |
| Recombinant human TNF-α | #AF-300-01A | PeproTech |
| Falcon 5 mL round bottom polystyrene test tube, with cell strainer snap cap  (35 µm nylon mesh) | # 352235 | Corning |
| 1.5 mL capless graduated microcentrifuge tube | #NC-509-GRD-Q | Quality Scientific Plastics |
| 24-well suspension plate | #662102 | Greiner |
| 0.01% (w/v) poly-l-lysine solution | #P4707 | Sigma-Aldrich |

**Antibodies used for flow cytometry**

| **Items** | **Catalog No.** | **Company** | **Clone** |
| --- | --- | --- | --- |
| True Stain Monocyte Blocker | #426101 | BioLegend |  |
| Mouse anti-human CD16 antibody-FITC conjugate | #555406 | BD Biosciences | 3G8 |
| Mouse anti-human CD11b antibody-APC-Cy7 conjugate | #560914 | BD Biosciences | ICRF44 |
| Mouse anti-human CD66b antibody-PerCP-Cy5.5 conjugate | #562254 | BD Biosciences | G10F5 |
| Sytox Orange | #S-11368 | Molecular Probes |  |
| Mouse anti-human MPO antibody–  FITC conjugate | #ab11729 | Abcam | 2C7 |
| Mouse IgG1κ-FITC conjugate | #555909 | BD Biosciences | MOPC-21 |
| Mouse IgG1κ–APC-Cy7 conjugate | #557873 | BD Biosciences | MOPC-21 |
| Mouse IgMκ–PerCP-Cy5.5 conjugate | #560857 | BD Biosciences | G155-228 |

**Reagents for reverse transcription-quantitative PCR**

| **Reagents** | **Catalog No.** | **Company** |
| --- | --- | --- |
| TRIzol | #15596-018 | Invitrogen |
| RNAlater Stabilization Solution | #AM7020 | Invitrogen |
| DNase I | #18068 | Invitrogen |
| SuperScript VILO cDNA synthesis kit | #11754250 | Invitrogen |
| EagleTaq Master Mix | #5529085190 | Roche |

**Taqman probes for reverse transcription-quantitative PCR**

| **Target gene** | **Catalog No.** |
| --- | --- |
| *Tnf-α* | Mm00443260_g1 |
| *Il-10* | Mm00439614_m1 |
| *Nos2* | Mm00440502_m1 |
| *Arg-1* | Mm00475988_m1 |
| *Hif-1α* | Mm00468869_m1 |
| *Vegf-a* | Mm01281447_m1 |
| *Plgf* | Mm00435613_m1 |
| *Myod1* | Mm01203489_g1 |
| *Myog* | Mm00446195_g1 |
| *18S rRNA* | Mm03928990_g1 |

Taqman probes used in reverse transcription-quantitative PCR were purchased from Applied Biosystems.

**Reagents for histological assessment**

| **Reagents** | **Catalog No.** | **Company** | **Clone** |
| --- | --- | --- | --- |
| Tissue-Tek O.C.T. Compound | #4583 | Sakura Finetek, Tokyo, Japan |  |
| Anti-histone H3 (citrulline R2+R8+R17) antibody-ChIP Grade | #ab5103 | Abcam |  |
| Goat anti-rabbit IgG (H+L) highly cross-adsorbed secondary antibody, Alexa Fluor 488 | #A-11034 | Invitrogen |  |
| Target Retrieval Solution | #S1699 | DAKO |  |
| Protein Block Serum-Free | #X0909 | DAKO |  |
| Rabbit polyclonal anti-mouse fibrinogen antibody | #ab27913 | Abcam |  |
| Rat anti-mouse CD31 antibody | #550274 | BD Biosciences | MEC13.3 |
| Purified rat IgG control | #6-001-A | R&D systems |  |
| Goat anti-rat IgG (H+L) cross-absorbed secondary antibody, Alexa Fluor 594 | #A-11007 | Invitrogen |  |
| Avidin/biotin blocking kit | #SP-2001 | Vector Labs |  |
| Isolectin GS-IB4 from *Griffonia simplicifolia*, biotin–XX conjugate | #121414 | Molecular Probes |  |
| Streptavidin, Alexa Fluor 488 conjugate | #S11223 | Invitrogen |  |
| Anti-α-smooth muscle actin (αSMA)–Cy3 antibody | #C6198 | Sigma-Aldrich | 1A4 |
