# Supplementary material for: Batroxobin accelerated tissue repair via neutrophil extracellular trap regulation and defibrinogenation in a murine ischemic hindlimb model
Source: PLoS One. 2019 Aug 16;14(8):e0220898. doi: 10.1371/journal.pone.0220898 (PMC6697371; doi:10.1371/journal.pone.0220898)
Supplement: S3 Table — (DOCX) [file pone.0220898.s003.docx]

**S3 Table. Relative gene expression in ischemic ATM of the DF-521-administered group vs. the control (Fig. 3).**

|  | Day 3 | Day 7 |
| --- | --- | --- |
| *Tnf-α* | 0.311 ± 0.0914* | 0.655 ± 0.3510* |
| *Il-10* | 0.493 ± 0.2077* | 1.190 ± 0.3415 |
| *Nos2* | 0.379 ± 0.2601** | 0.391 ± 0.0512* |
| *Arg-1* | 0.224 ± 0.2609* | 4.033 ± 1.7980* |
| *Hif-1α* | 0.331 ± 0.1326* | 0.739 ± 0.0872* |
| *Vegf-a* | 0.191 ± 0.3856** | 0.165 ± 0.0414* |
| *Plgf* | 0.349 ± 0.0926* | 1.397 ± 0.2235** |
| *Myod1* | 0.576 ± 0.5416 | 0.834 ± 0.2780 |
| *Myog* | 0.293 ± 0.1064* | 1.846 ± 0.4252* |

The values present the fold change of the expression of each gene as mean ± SD. Asterisks indicate statistical significance. **P* < 0.05, ***P* < 0.01; n = 4 or 5.
