# Supplementary figures and images for: Batroxobin accelerated tissue repair via neutrophil extracellular trap regulation and defibrinogenation in a murine ischemic hindlimb model
Source: PLoS One. 2019 Aug 16;14(8):e0220898. doi: 10.1371/journal.pone.0220898 (PMC6697371; doi:10.1371/journal.pone.0220898)

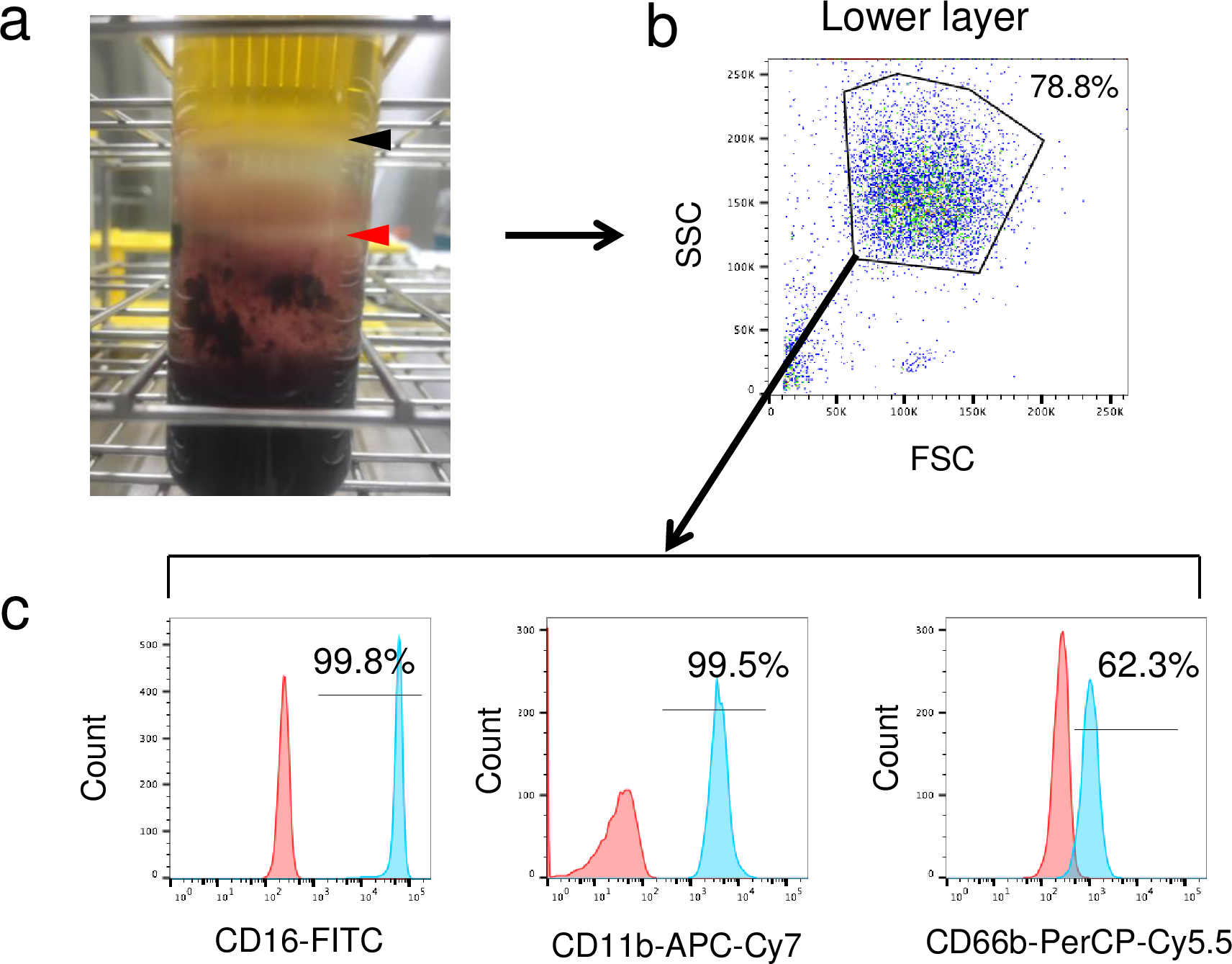

Supplement: S1 Fig — (TIF) [file pone.0220898.s004.tif]

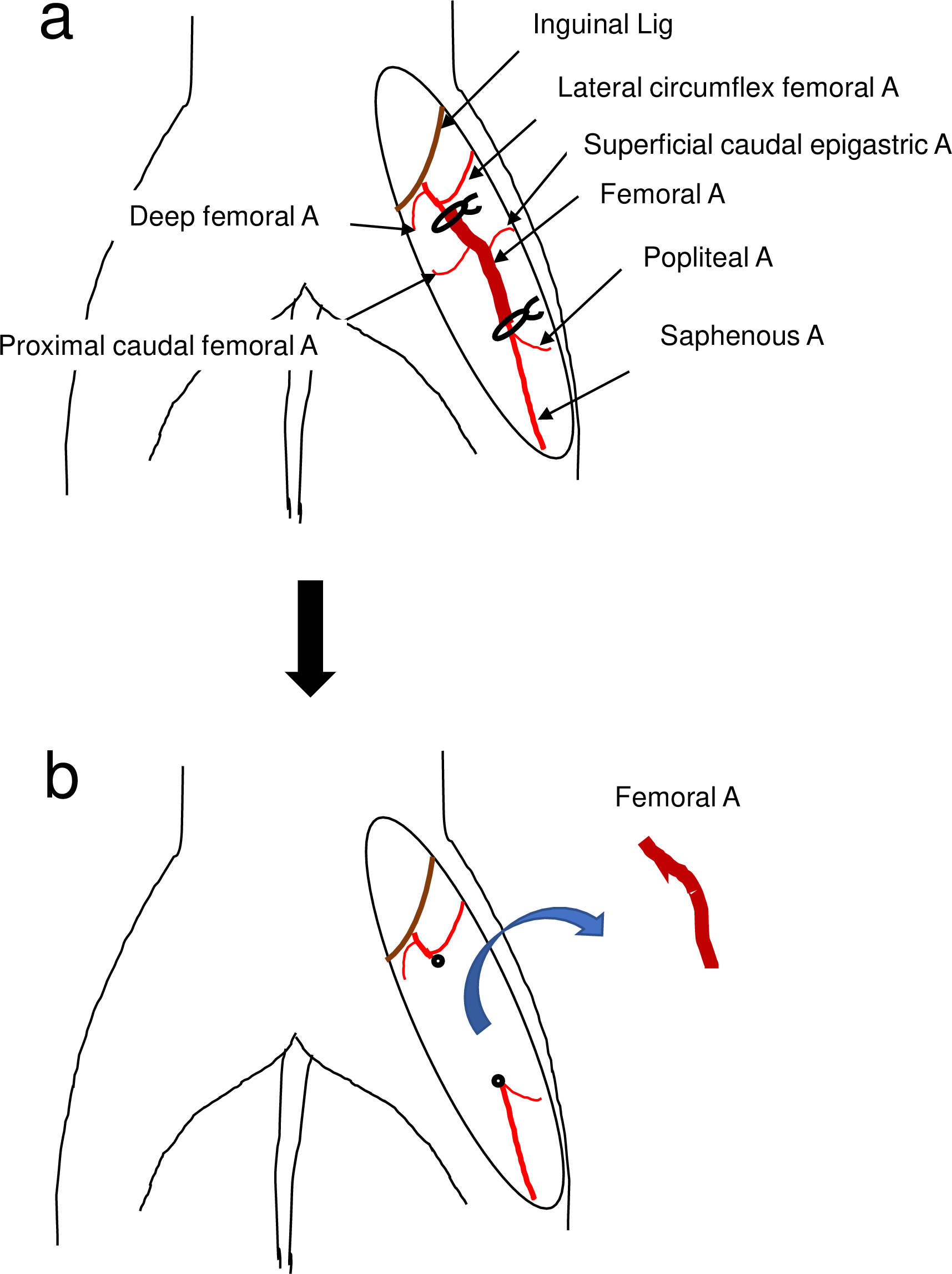

Supplement: S2 Fig — (TIF) [file pone.0220898.s005.tif]

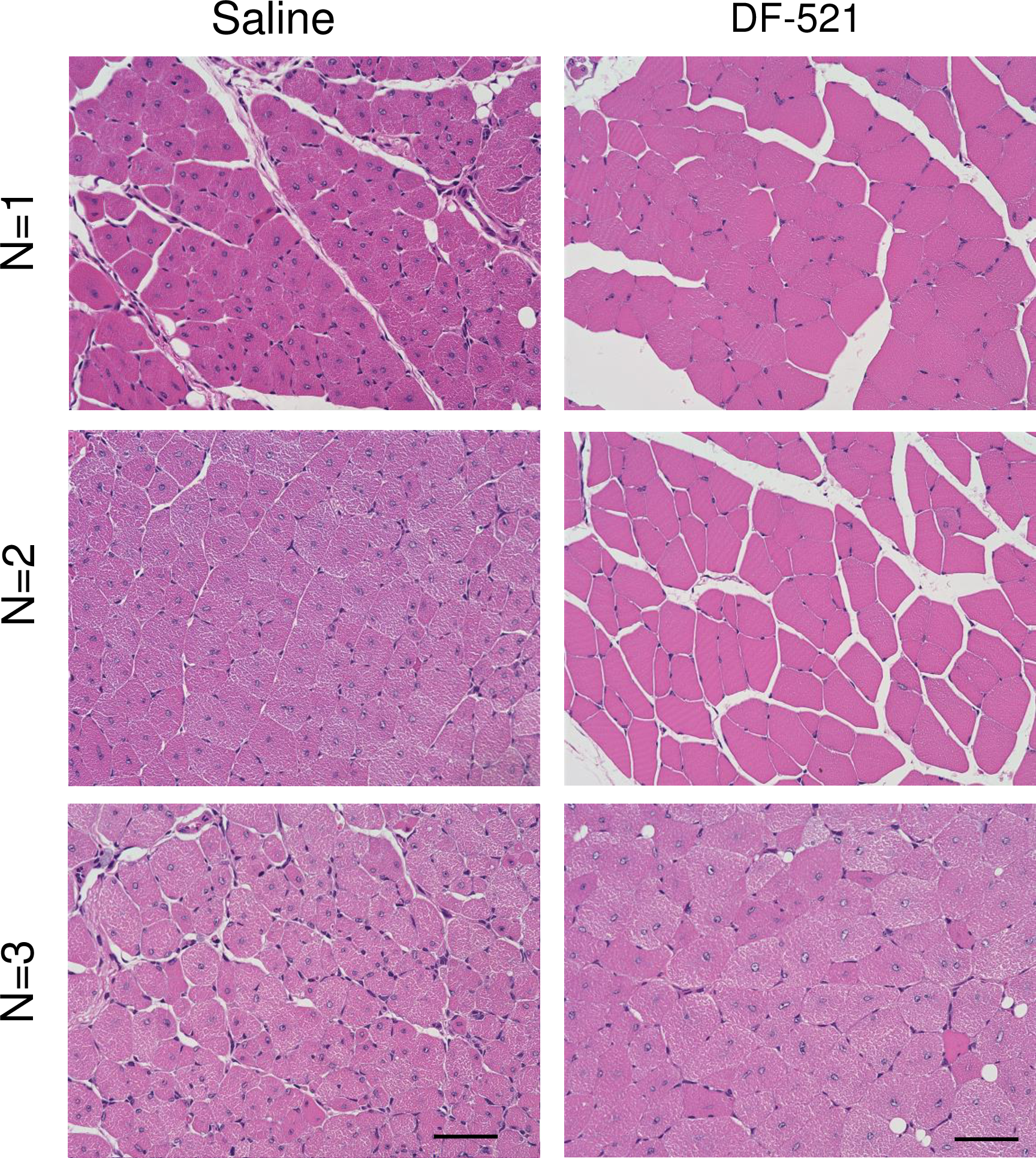

Supplement: S3 Fig — (TIF) [file pone.0220898.s006.tif]
